# Progress in Surgery of the Intestines

**Published:** 1901-08-31

**Authors:** 


### Progress in Surgery of the Intestines.

**Duodenal Ulcer,** says Marocco,[1] is usually single, and generally occurs in the first part of the duodenum about three to eight centimetres beyond the pylorus. The ulcer is circular or elliptical and situated in the long axis of the bowel. It is more liable to perforate than gastric ulcer, and men are seven or eight times more liable to it than women. The disease is chronic, lasting eight or ten years or more. The presence of pain in the right superior quadrant of the abdomen, radiating to the shoulder and tenth and twelfth dorsal vertebræ, coming on two or three hours after food, and occasional vomit containing small quantities of bile, with undigested muscle tissue in the fæces, suggest duodenal rather than gastric ulcer. Melæna is more common than hæmatemesis. If prolonged trial of medical treatment is not successful, and the patient is wasting, operative measures should be undertaken. And of these gastro-enterostomy is the operation to be selected. This was the operation performed in two cases by the author. Weir[2] also contributes a paper with clinical notes and analyses of the 51 cases of operation for its relief already published. In 25 of these cases the lesion was recognised and closed at the operation: of these 13 underwent operation after 30 hours' delay and all died; one case, however, recovered after a delay of nine days before operation, but here the perforation had formed a chronic abscess and sinus; 12 others were operated on within that time; 8 survived, giving thus only 33⅓ per cent. mortality. In 26 cases a wrong diagnosis was made and the ulcer was not found; in 12 cases the ulcer was found though the previous diagnosis was wrong; and in 13 cases the diagnosis was approximately right and the ulcer was found. Abbe[3] reports a case of universal peritonitis following ruptured duodenal ulcer in which operation was performed 27 hours later, and the patient recovered. He thinks that if an operation was done within the first six hours about 25 per cent. of these cases could be saved. Lowson[4] also reports a case of ruptured duodenal ulcer treated successfully by operation.

**Meckel's diverticulum** is an occasional cause of intestinal obstruction. Erdmann[5] records a case in which a large diverticulum was found adherent to the appendix and strangulating the bowel. The appendix was removed, and the patient made a good recovery. Another case is recorded by Campbell[6] in which a man of seventy years of age died of obstruction, which at the post-mortem examination was found to be due to a Meckel's diverticulum, and Taylor[7] reports another upon which an operation was unsuccessfully performed. In the last case the diverticulum came off within two inches of the ileo-cæcal valve.

**Cancer of the Large Intestine,** says Lardennois,[8] is attended by the following symptoms: 1. In the early stages paroxysmal attacks of colic occur, with periods of prolonged constipation, followed by diarrhœa. 2. Progressive emaciation in spite of properly-conducted treatment. 3. The presence of a tumour, especially if it be found to vary in size and consistence at different examinations. If there is a strong probability of the presence of a growth, exploratory laparotomy should always be undertaken. The duration of cases of cancer of the larger intestine averages one year, death usually taking place from some mechanical or infective complication. The judicial operation is enterotomy, followed, wherever possible, by circular enterorraphy. The palliative operations are colostomy, anastomosis, and exclusion. Colostomy has the strong objection of leaving an artificial anus, and anastomosis or exclusion are, therefore, better. Anastomosis according to the extent and seat of the disease, may be a cæco-colostomy, colo-colostomy, ileo-colostomy, or entero-rectostomy. The last is frequently necessary, since the growth often occurs in the sigmoid or recto-sigmoid region. The difficulty in its performance of applying sutures low down in the pelvis has caused the author to use a modified form of Murphy button. Exclusion consists in separating the cancerous from the healthy intestine and restoring the continuity by circular enterorraphy. It is a resection without ablation of the growth, and presents no advantages over entero-rectal anastomosis. Heaton[9] reports a successful case of excision of the cæcum, vermiform appendix, and portion of the ileum for carcinoma, and a somewhat similar case is reported by Morton.[10]

**Enterectomy and Intestinal Anastomosis.** — Barker[11] discusses the limitations of enterectomy. Taking 1 metre (39·5 inches) as a minimum limit he has been able to collect 27 successful resections of intestine exclusive of his own. These range themselves as follows: Between one and two metres, 20 cases; between two and three metres, five cases; and between three and four metres, two cases. The largest amount of intestine removed was 330 centimetres, or 10 ft. ½. in., from a boy aged eight years, whose condition a year later showed no evidence that the loss of so much of his small intestine had unfavourably affected his health. From all this it would appear that about two metres of small intestine, say 6 ft., may be removed without any serious damage to the subsequent assimilative powers. It is probable that the resection is better tolerated the lower down the intestinal tract it is done. O'Hara[12] proposes a method of performing anastomosis of the hollow viscera by a new instrument, and claims for it the possession of the following advantages:—(1) The wide application of a single instrument; for with the same instrument one may do a resection of the pylorus, of the cæcum, and of the small and large intestines. (2) Anastomosis can also be performed on any of the hollow viscera, including the large and small intestines, stomach, and even intestines of unequal calibre; the various gall-bladder operation can also be performed. (3) The dangers of fæcal matter escaping into the peritoneal cavity are prevented, as the bowel remains closed until the very last moment. (4) The speediness of the method is not at the expense of accuracy. (5) The danger of secondary stricture of the bowel is reduced to a minimum. (6) No foreign body is left in the intestinal canal. The instrument consists of two pairs of straight forceps the jaws of which are very slender and $2\frac{1}{2}$ inches long for ordinary work; for special work they can be made longer. Instead of being made roughened as in ordinary hæmostatic forceps, they are grooved down the centre of one blade; the opposite one has a ridge, similar to a pile clamp; both forceps are held together by means of an adaptation of the serrefine. In making a resection followed by end-tc-end anastomosis, the serrefine is removed and each forceps is placed transversely across the bowel at the point selected to mark the upper and lower borders of the resection and locked. The intervening portion of bowel is then cut away, the forceps brought together and locked by the serrefine and the bowel united. The forceps are then unclamped and removed, the remaining opening being closed by one stitch. Lee[13] has also devised an intestine holder for facilitating the end-to-end suture of intestine, for which he claims the following advantages: 1. One instrument will fit all sizes of intestine. 2. Any method of suture may be used. 3. There is no necessity for any retention sutures. 4. No assistants are necessary to complete the suture. 5. There is very little tendency to diaphragm formation during and after suture. The instrument consists primarily of two pairs of pivotal arms forming, when extended, what might be termed a double cross, and adapted, when in this position, to have the cut ends of the intestine extended over them. The outer surfaces of the arms, which come in contact with the intestine, are serrated in order to prevent any movement of the intestine after it has been placed on the instrument in position. These arms are adapted to be separated in order to fit any sized intestine, or to give any required tension or relaxation to the intestine during the suture. Cases in which the Murphy button was used are reported by Todd,[14] Newbolt,[15] and Bonnin.[16]

**Intussusception.**—Michaux[17] insists on the necessity of resorting to surgical methods in the treatment of this affection. Of two of these methods, the establishment of a false anus and simple disinvagination, he says little, as they are applicable only under infrequent conditions. The former should not be practised except when the patient is collapsed and almost moribund, and the latter, which, when it can be practised with safety, is by far the most successful procedure, is unfortunately too often rendered impossible by the existence of adhesions and the presence of an abdominal tumour. The two remaining methods resection of the whole of the involved portion of intestine and resection only of the invaginated portion, Michaux advocates the latter. The former he acknowledges to be a more radical procedure, and he is disposed to regard it as the operation of the future; but though the statistics of its mortality have recently been less unsatisfactory, it must at present be regarded as a dangerous and difficult method of surgical treatment. Erdmann[18] says that in 30 per cent. of all cases of acute obstruction we find intussusception as the cause, and fully 50 per cent. of all cases of intussusception occur in children under 10 years of age; of these, again, 50 per cent. occur under the age of 12 months. Kammerer[19] believes that of the non-operative methods of treatment of intussusception the only one deserving of any serious attention is the injection of fluids into the rectum, and this is only applicable in the intussusceptions of the large intestine; in acute cases it should not be attempted after the first twenty-four hours. The most serious objection to its use is our inability to recognise that reduction has really been accomplished. After one failure laparotomy is indicated, and in very acute cases the method should not be employed at all. Gibson[20] has analysed 187 laparotomies for acute intussusception: of these the tumour was reducible in 126 cases, with a mortality of 36 per cent., while the mortality of the irreducible cases was 64 per cent., and of the gangrenous 95 per cent. In 34 cases in which an artificial anus was established the mortality was 83 per cent., and of 32 resections 81 per cent. He concludes that in order to obtain success operations must be performed early enough to find the intussusception reducible—that good results depend entirely on reducibility. Cases of laparotomy for intussusception are reported by Knaggs,[21] Kerr,[22] and Tymms.[23]

[1] Brit. Med. Jour., Jan. 26, 1901. Ep., No. 62. [2] Brit. Med. Jour., Jan. 19, 1901. Ep., No. 46, [3] Med. Rec., March 2, 1901, p. 352. [4] Brit. Med. Jour., May 25, 1901, p. 1,273. [5] Med. Rec., Nov. 3, 1900, p. 716. [6] Brit. Med. Jour., May 25. 1901, p. 1263. [7] Ibidem, April 6, 1901, p. 827 [8] Med. Chron., Feb. 1901, p. 373. [9] Lancet, March 30, 1901, p. 928 [10] Brit. Med. Jour., April 13, 1901, p. 878. [11] Lancet, April 17, 1901, p. 1,189. [12] Annals of Surgery, Feb. 1901, p. 179. [13] Annals of Surgery, Jan. 1901, p. 23. [14] Austral. Med. Gaz., March 20, 1901, p. 89. [15] Lancet, May 11, 1901 p. 1,333. [16] Austral. Med. Gaz., March 20, 1901, p. 90. [17] Brit. Med. Jour., Jan. 26. 1901. Ep. No. 63. [18] Med. Chron., Jan. 1901, p. 298. [19] Ibidem, p. 299. [20] Ibidem, p. 300. [21] Lancet, Dec. 1, 1900, p. 1,573. [22] Brit. Med. Jour., Nov. 24, 1900, p. 1,499. [23] Brit. Med. Jour., Feb. 9, 1901, p. 341.

